# Epidural analgesia and avoidance of blood transfusion are associated with reduced mortality in patients with postoperative pulmonary complications following thoracotomic esophagectomy: a retrospective cohort study of 335 patients

**DOI:** 10.1186/s12871-019-0832-5

**Published:** 2019-08-22

**Authors:** Kai B. Kaufmann, Wolfgang Baar, Torben Glatz, Jens Hoeppner, Hartmut Buerkle, Ulrich Goebel, Sebastian Heinrich

**Affiliations:** 1 Department of Anesthesiology and Critical Care Medicine, University of Freiburg, – University of Freiburg, Hugstetter Strasse 55, 79106 Freiburg, Germany; 2 Department of General and Visceral Surgery, Medical Center - University of Freiburg, Faculty of Medicine – University of Freiburg, Hugstetter Strasse 55, 79106 Freiburg, Germany

**Keywords:** Esophagectomy, Postoperative pulmonary complications, Thoracic epidural anesthesia, Blood transfusion, Independent risk factors, 90-days mortality

## Abstract

**Background:**

Postoperative pulmonary complications (PPCs) represent the most frequent complications after esophagectomy. The aim of this study was to identify modifiable risk factors for PPCs and 90-days mortality related to PPCs after esophagectomy in esophageal cancer patients.

**Methods:**

This is a single center retrospective cohort study of 335 patients suffering from esophageal cancer who underwent esophagectomy between 1996 and 2014 at a university hospital center. Statistical processing was conducted using univariate and multivariate stepwise logistic regression analysis of patient-specific and procedural risk factors for PPCs and mortality.

**Results:**

The incidence of PPCs was 52% (175/335) and the 90-days mortality rate of patients with PPCs was 8% (26/335) in this study cohort. The univariate and multivariate analysis revealed the following independent risk factors for PPCs and its associated mortality. ASA score ≥ 3 was the only independent patient-specific risk factor for the incidence of PPCs and 90-days mortality of patients with an odds ratio for PPCs being 1.7 (1.1–2.6 95% CI) and an odds ratio of 2.6 (1.1–6.2 95% CI) for 90-days mortality. The multivariate approach depicted two independent procedural risk factors including transfusion of packed red blood cells (PRBCs) odds ratio of 1.9 (1.2–3 95% CI) for PPCs and an odds ratio of 5.0 (2.0–12.6 95% CI) for 90-days mortality; *absence of* thoracic epidural anesthesia (TEA) revealed the highest odds ratio 2.0 (1.01–3.8 95% CI) for PPCs and an odds ratio of 3.9 (1.6–9.7 95% CI) for 90-days mortality.

**Conclusion:**

In esophageal cancer patients undergoing esophagectomy via thoracotomy, epidural analgesia and the avoidance of intraoperative blood transfusion are significantly associated with a reduced 90-days mortality related to PPCs.

## Introduction

Esophagectomy for esophageal cancer treatment is associated with increased rates of up to 50% for postoperative morbidity and of 12% for postoperative mortality [1–4]. Postoperative pulmonary complications (PPCs) represent the most frequent adverse events after esophagectomy with an incidence of up to 38% affecting patients’ short- and long-term outcome [1]. PPCs are the major cause of early death after esophagectomy, especially when the transthoracic approach via thoracotomy was taken [1, 5, 6]. Several risk factors for PPCs after esophagectomy have been identified. These are increased age, female gender, preoperative comorbidities (diabetes, arterial hypertension, chronic obstructive lung disease), neoadjuvant radio- or chemotherapy, low preoperative forced expiratory volume in 1 s (FEV_1_), low diffusion capacity of the lung for carbon monoxide (DLCO), smoking, transthoracic resection, high ASA score, high amounts of intraoperative fluids and increased blood loss [1, 7–9]. Whereas most of the risk factors have to be considered as non-adjustable, current research focuses on procedural risk factors that may be optimized. According to current literature it appears evident that intraoperative fluid overload is one of the major risk factors for PPCs and mortality after esophagectomy [10–12]. However, data on the quality of intraoperative fluids (for example colloids, PRBCs, FFPs) and its influence on PPCs and the subsequent mortality are scarce [13]. TEA is another procedural and anesthesia-related factor potentially reducing the incidence of PPCs after esophagectomy. Results on this topic appear contradictory [2, 14–20]. Although it has been shown that perioperative TEA in patients after esophagectomy significantly decreases time on ICU, there is no study that provides data on superior oncological results and mortality decrease among patients with PPCs due to TEA [19]. De la Gala and colleagues showed that the use of sevoflurane instead of propofol for anesthesia maintenance in lung surgery with thoracotomy led to a decrease of PPCs [21]. To our best knowledge there is no study that examined this effect in esophageal cancer patients undergoing esophagectomy.

The aim of this study was to identify further modifiable risk factors and to validate existing risk factors for mortality related to PPCs after esophagectomy in esophageal cancer patients. This is the first study that focuses on mortality related to PPCs after esophagectomy.

## Methods

**Fig. 1:**
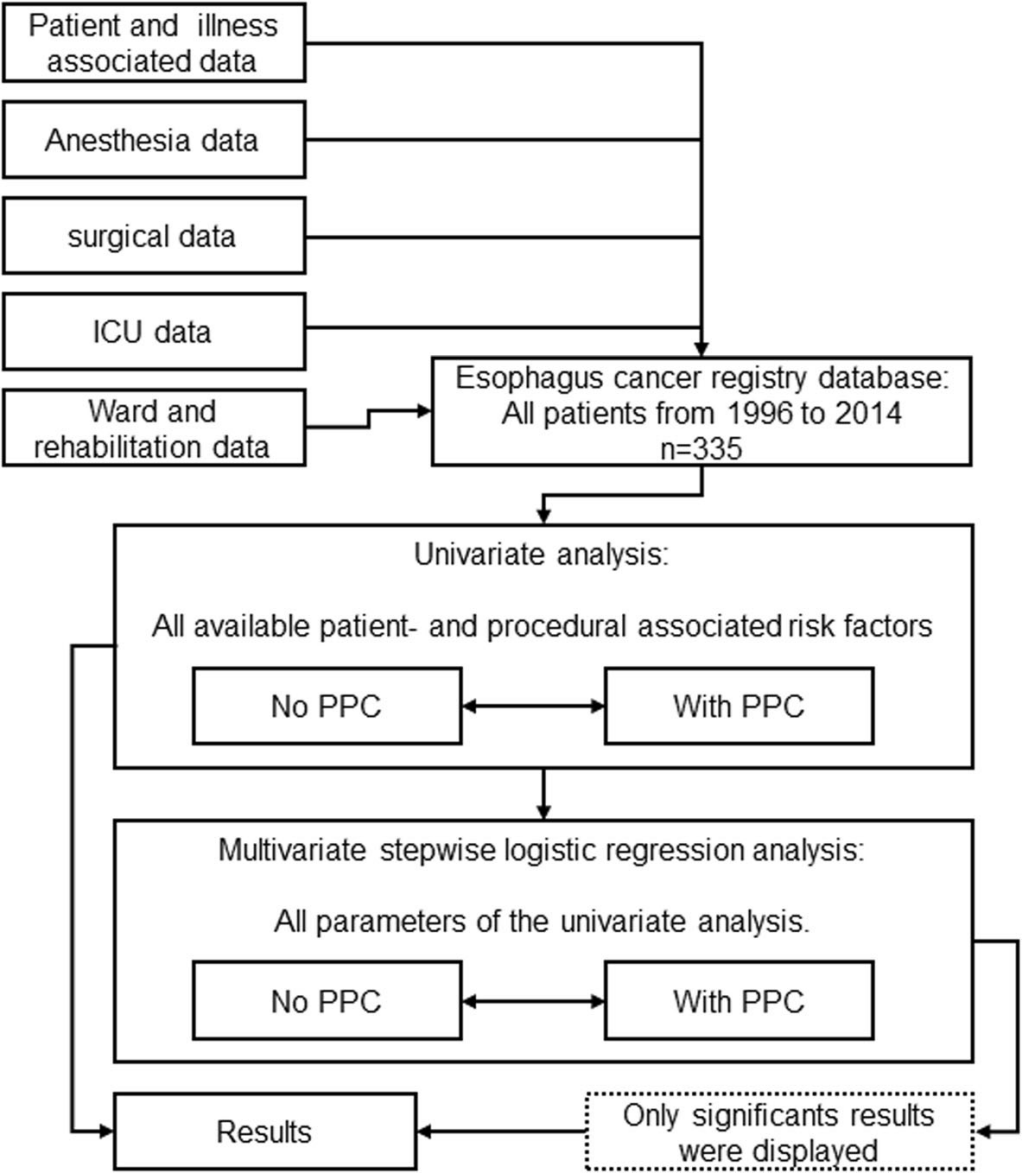
Case selection. ICU = Intensive care unit; PPCs = Postoperative pulmonary complications

This retrospective cohort study was approved by the local Ethics Committee, University of Freiburg, Germany (EK569/14 December 9th 2014). The study was conducted at the Department of Anesthesiology and Intensive Care and the Department of General and Visceral Surgery, University Medical Center, Freiburg, Germany. The study was planned and designed in accordance with the initiative for Strengthening the Reporting of Observational Studies in Epidemiology STROBE, using the suggested checklist for epidemiological cohort studies [22]. Patients’ data were only entered into the cancer registry, if formal informed consent was obtained. In this retrospective cohort study 335 esophageal cancer patients, who underwent open esophagectomy between January 1st 1996 and March 31st 2014, were analyzed. A priori sample size calculation was not applicable due to the retrospective study design. Figure 1 shows the underlying data collection and statistical process of this study.

### Surgery

The majority of patients underwent open esophagectomy via the Ivor-Lewis thoraco-abdominal approach. This included a right-sided thoracotomy and a median laparotomy. Only 26 (8%) patients had a transhiatal esophagectomy via laparotomy. For reconstruction the formation of a pulled-up gastric tube was the preferred technique, only 14 (4%) patients underwent colon interposition.

### Anesthesia

The intraoperative anesthesia management was not standardized by study protocol. Whenever feasible a combination of thoracic epidural and general anesthesia was used. The epidural pain catheter was placed at interspaces T4/5, T5/6 or T6/7 prior to general anesthesia. After negative test dose injection epidural analgesia was induced by injection of a total of 8–10 ml of ropivacaine 0.2% plus epidural sufentanil (0.2–0.3 μg/kg) or fentanyl (2–3 μg/kg), followed by a continuous epidural infusion of ropivacaine 0.2% and sufentanil (0.5 μg/ml) or fentanyl (5 μg/ml) at 8 ml/h during surgery until the third postoperative day. A total of 49 patients (49/335, 15%) did not have an epidural pain catheter. These patients were treated with a systemic opioid therapy. The postoperative pain control for patients without epidural analgesia followed a standardized procedure: On ICU, analgesia was provided nurse controlled by using intravenous and if possible enteral opioids. If patients were transferred from ICU to the normal care ward and standard oral opioid analgesia was insufficient, an intravenous patient-controlled analgesia system was established. These patients were visited daily by the institutional acute pain service. In 24 patients (24/49, 49%) the placement of the epidural pain catheter was described as unsuccessful. In the remaining 25 patients, epidural analgesia was not provided because of patient refusal.

Mostly, anesthesia was maintained by isoflurane, desflurane or sevoflurane. The minority of patients had total intravenous anesthesia with propofol. Patients´ radial artery was cannulated for continuous blood pressure and intermittent blood gas monitoring.

For endotracheal intubation with the double-lumen tube muscle relaxant was used. During surgery depth of muscle relaxation was continuously monitored. Adequate muscle relaxation was defined as 1 twitch out of 4 in the train-of-four technique. If patients were extubated in the operation room muscle relaxation was completely reversed by using neostigmine until four equal twitches were present. In all patients a left sided double-lumen endobronchial tube (35–39 Fr/Ch (11.7–13 mm)) was used. Positioning of the double-lumen endotracheal tube was checked visually by fiberoptic bronchoscope. The ventilator settings were standardized in accordance with an in-house standard operating procedure for single-lung ventilation. Pressure-controlled ventilation with a positive endexspiratory pressure of 5–7 cm H_2_O and peak pressure less than 30 cm H_2_O for both, double-lung and single-lung ventilation was chosen. Inspiratory oxygen fraction was set as low as possible to avoid hypoxia with a peripheral oxygen fraction above 90%. This resulted in an oxygen fraction of 0.4 for double-lung and usually 0.8 for single-lung ventilation. Lung protective ventilator settings were applied. These included tidal volumes up to 8 ml/kg and a respiratory frequency of 10–15/min for double-lung ventilation, while for single-lung ventilation tidal volumes of 6–7 ml/kg with a respiratory frequency of 10–15 /min were applied. Re-inflating of the previously non-ventilated lung was restarted by a manual recruitment manoeuvre for 10 s to 30 cm H_2_O five times. Norepinephrine, balanced electrolyte solutions (either Jonosteril® [Fresenius Kabi, Bad Homburg Germany] or Normofundin® [Braun, Melsungen Germany]) and hydroxyethyl starch 6% [Voluven® or Volulyte® [Fresenius Kabi, Bad Homburg Germany] were used to keep the arterial blood pressure within physiological ranges. According to the in-house standard operating procedure a hemoglobin level below 8 g/dl was considered as a threshold for PRBC administration during surgery. Intraoperative fluid administration was extracted from anesthetic protocols.

### Definition of PPCs

All PPCs were defined according to the International Consensus on Standardization of Data Collection for Complications Associated with Esophagectomy (*Esophagectomy Complications Consensus Group (ECCG))* [23]. The Re-intubation rate was documented. The indication for re-intubation was hypercapnia or hypoxia. Hypercapnia was defined as an arterial CO_2_ partial pressure leading to a decrease of pH below 7,25 or unconsciousness. Hypoxia as an indication for re-intubation was defined as an arterial O_2_ partial pressure below 55 mmHg. Pleural effusion was defined as new chest-tube insertion in addition to those placed in the operation room or delayed removal of intraoperatively placed chest tubes after the 10th postoperative day. The rate of postoperative chylothorax and pleural empyema was also defined according ECCG. Tracheostomy was performed due to prolonged respiratory weaning either after re-intubation or because the initial placed endotracheal tube in the OR could not be removed. Pneumonia was defined as new pulmonary infiltrate on chest X-ray with associated leukocytosis, fever, new purulent sputum, need for antibiotic therapy and increased oxygen demand via face mask. The assessment of chest X-rays was performed by two independent radiologists. We collected all parameters by chart review to diagnose postoperative pneumonia [23].

### Statistical analysis

For parameters that were distributed normally, mean value and standard deviation were calculated. Median and interquartile range were calculated due to the fact that parameters were not normally distributed. Univariate statistical analysis was performed by dividing the cohort into two groups, with and without PPCs. Statistical analyses of continuous variables were calculated using the Mann-Whitney U test for not-normally distributed parameters and the Students T-test was used for normally distributed values. Categorical variables were analyzed with χ2 test. All parameters included in the univariate analysis were used for the multivariate stepwise logistic regression analysis to assess the impact of these on the incidence of PPCs and 90-days mortality of patients with PPCs. Continuous variables were dichotomized. The following parameters were included in the multivariate stepwise logistic regression analysis: Surgical approach (transhiatal versus thoracoabdominal); reconstruction (gastric tube versus colon interponate); duration of surgery ≥430 min (3. quartile of the study cohort); blood loss ≥1000 ml; norepinephrine ≥0.07 μg/kg/min (3. quartile of the study cohort); total amount of intraoperative crystalloids ≥7000 ml (3. quartile of the study cohort); intraoperative use of colloids, packed red blood cells, fresh frozen plasma; epidural anesthesia; total intravenous anesthesia versus gas to maintain intraoperative general anesthesia; age ≥ 65 years; gender; alcohol abuse; nicotine dependency; neoadjuvant therapy; ASA score ≥ 3 or 4; BMI ≤ 18.5; UICC grade ≥ 3 or 4; hemoglobin level preoperative ≤11 mg/dl; extubation in the OR. The significant results of the multivariate stepwise logistic regression analysis are highlighted. A *P* value of ≤0.05 was considered statistically significant. IBM SPSS Statistics for Windows, (Version 23.0 Armonk, NY USA: IBM Corp.) was used for statistical analysis.

## Results

Table 1 highlights the results of patient-specific and procedural (surgery- and anesthesia-related) parameters of the entire study cohort. The majority of patients had neoadjuvant therapy 78% (262/335), most of them were male 87% (290/335) and underwent thoracoabdominal esophagectomy 92% (309/335) reconstructed by gastric tube 96% (321/335). Apart from that, most patients had perioperative thoracic epidural anesthesia (TEA) 85% (286/335). The overall 30- and 90-days mortality rate in this study cohort was 5% (17/335) and 11% (37/335). The 90-days-mortality rate of patients with PPCs was 8% (26/335). One hundred seventy-five patients (52%) developed PPCs. The most frequent complication was pneumonia 34% (114/335), followed by pleural effusion 31% (104/335), re-intubation 20% (66/335) and tracheostomy 12% (39/335). Pleural effusion was diagnosed in 104 patients, of these 51 patients received a new chest tube after surgery. Twenty-three patients (23/335, 7%) developed a chylothorax, whereas 18 patients (18/335, 5%) showed postoperative pleural empyema. A total of 66 patients (66/335, 20%) were re-intubated. Of these, 37 patients (37/66, 56%) had a tracheostomy in their further clinical course. Tracheostomy was performed in a total of 39 patients. Of these, 37 (37/39, 95%) were re-intubated on ICU and only 2 had a tracheostomy due to delayed weaning from the endotracheal tube placed in the operation room. The total amount of PPCs differs from the amount of patients with PPCs as some patients suffered from more than one PPC. As PPCs are linked to each other and the occurrence of one predisposes for another, the numbers highlighted in the following tables refer to the number of patients.

**Table 1:** Baseline patient-specific and procedural characteristics of the entire cohort

	Entire cohort (*n* = 335)
Patient-specific parameters	
Age (years)	62 ± 10
Gender (male/female)	290 (87%)/ 45 (13%)
Alcohol abuse	117 (35%)
Smoking	170 (51%)
Neoadjuvant RCT	262 (78%)
ASA 1/ 2/ 3/ 4 (n)	10 (3%)/183 (55%)/137 (41%)/5 (2%)
BMI (kg/m^2^)	24 (5)
UICC I/ II	251 (75%)
UICC III/ IV	84 (25%)
Procedural parameters	
Surgery-related	
Surgical approach	
Transhiatal	26 (8%)
Thoracoabdominal	309 (92%)
Reconstruction	
Gastric tube	321 (96%)
Colon interposition	14 (4%)
Duration of surgery (hours)	7.0 (2.2)
Blood loss (ml)	700 (600)
Anesthesia-related	
Average Norepinephrine (μg/kg/min)	0.03 (0.07)
Crystalloids (ml)	5500 (3000)
FFP (ml)	136 ± 760
PRBC (ml)	426 ± 1188
Colloids (ml)	1000 (500)
TEA	286 (85%)
TIVA/Gas	94 (28%) / 240 (72%)
Isoflurane	188 (56%)
Desflurane	50 (15%)
Sevoflurane	2 (0.6%)
Hospital stay (days)	22 (19)
ICU stay (days)	8 (7)
30-days-mortality overall	17 (5%)
90-days-mortality overall	37 (11%)
Pneumonia	114 (34%)
Pleural effusion	104 (31%)
New postoperative thoracic drainage	51 (15%)
Chylothorax	23 (7%)
Pleural empyema	18 (5%)
Re-intubation	66 (20%)
Tracheostomy	39 (12%)
Re-intubation followed by tracheostomy	37 (11%)
PPCs	175 (52%)
90-days-mortality with PPCs	26 (8%)

Data are presented as number of patients (percentage), median (interquartile range) or mean (standard deviation)

*RCT* Radio−/Chemotherapy, *ASA* American Society of Anesthesiology, *BMI* Body mass index, *UICC* Union Internationale Contre Cancer, *FFP* Fresh frozen plasma, *PRBC* Packed red blood cell, *TEA* Thoracic epidural anesthesia, *TIVA* Total intravenous anesthesia, *ICU* Intensive care unit, *PPCs* Postoperative pulmonary complications

Table 2 shows the results of the univariate analysis comparing patient-specific risk factors of patients with and without PPCs. Among patient-specific risk factors only the ASA score ≥ 3 showed a significantly higher rate in the PPC group (55/157 (35%) versus 87/178 (49%), *P* = 0.01)). All the other patient-specific risk factors including age, gender, alcohol abuse, smoking, neoadjuvant radio- and/or chemotherapy, body-mass-index (BMI), UICC (Union Internationale Contre Cancer) ≥3 and hemoglobin level before surgery showed no difference between patients with and without PPCs.

**Table 2:** Univariate analysis of patient-specific parameters of patients with and without PPCs

	No PPC (*n* = 157)	PPC (*n* = 178)	*P* Value
Patient-specific parameters			
Age (years)	61 ± 10	62 ± 10	0.1
Gender (male/female)	137 (87)/21 (13)	153 (86)/24 (13)	1
Alcohol abuse (n)	52 (33)	65 (37)	0.5
Smoking (n)	74 (47)	96 (54)	0.2
Neoadjuvant RCT (n)	124 (79)	138 (78)	1
ASA ≥ 3 (n)	55 (35)	87 (49)	**0.01**
BMI (kg/m^2^)	25 (22–27)	24 (22–28)	0.8
UICC ≥3 (n)	41 (26)	43 (24)	0.8
Hb before surgery (mg/dl)	12.7 (11.6–13.6)	12.6 (11.3–14)	0.65

Data are presented as number of patients (percentage), median (interquartile range) or mean (standard deviation)

*RCT* Radio−/Chemotherapy, *ASA* American Society of Anesthesiology, *BMI* Body mass index, *UICC* Union Internationale Contre Cancer, *Hb* Hemoglobin, *PPCs* Postoperative pulmonary complications

Table 3 shows the results of the univariate analysis comparing procedural risk factors of patients with and without PPCs. There were no differences with respect to surgery-related risk factors. Among anesthesia-related risk factors patients of the PPC group received a higher amount of crystalloids, more packed red blood cells (PRBCs) and less often TEA. To assess the importance of epidural analgesia among high risk patients with an ASA score ≥ 3 an additional analysis on this study cohort was conducted. Among patients with an ASA score ≥ 3 24 patients had no epidural analgesia. Of these, 8/24 patients developed PPCs and died within 90 days after surgery compared to 10 out of 118 patients who received preoperative epidural analgesia (*P* <  0.001).

**Table 3:** Univariate analysis of procedural parameters (surgery-related and anesthesia-related) of patients with and without PPCs

	No PPC (*n* = 157)	PPC (*n* = 178)	*P* Value
Procedural parameters			
Surgery-related			
Surgical approach (n)			
Transhiatal	10 (6)	16 (9)	0.42
Thoracoabdominal	148 (94)	161 (91)	
Reconstruction (n)			
Gastric tube	154 (98)	167 (94)	0.18
Colon interposition	4 (2)	10 (6)	
Duration of surgery (hours)	7 (6–8.3)	7.1 (6.2–8.5)	0.19
Blood loss (ml)	600 (300–1000)	750 (500–1000)	0.12
Anesthesia-related			
Norepinephrine (μg/kg/min)	0.03 (0–0.06)	0.02 (0–0.07)	0.9
Crystalloids (ml)	1100 (880–1924)	1404 (1008–2071)	**0.007**
Colloids (ml)	752 (73–1000)	710 (263–1035)	0.7
Colloids (n)	141 (90)	158 (89)	1
FFP (n)	19 (12)	21 (12)	1
PRBC (n)	44 (28)	78 (44)	**0.002**
TEA (n)	142 (90)	144 (81)	**0.019**
TIVA / Gas (n)	49 (31) / 108 (69)	45 (25)/ 132 (74)	0.27
Hospital stay (days)	17 (15–23)	30 (20–50)	**< 0.001**
Extubation on ICU	110 (70)	141 (79)	0.5
Pleural effusion	0	104 (58)	**< 0.001**
Chylothorax	0	23 (13)	**< 0.001**
ICU stay (days)	6 (5–8)	12 (7–26)	**< 0.001**

Data are presented as number of patients (percentage) or median (interquartile range)

*FFP* Fresh frozen plasma, *PRBC* Packed red blood cell, *TEA* Thoracic epidural anesthesia, *TIVA* Total intravenous anesthesia, *ICU* Intensive care unit, *PPCs* Postoperative pulmonary complications

The pain level at rest for the first three postoperative days did not show a difference between patients with and without PPCs. As a consequence patients with PPCs had a longer hospital and ICU stay. A total of 251 (75%) patients were postoperatively transferred to ICU with a retained one-lumen endotracheal tube. There was no difference with respect to the incidence of PPCs. There were 141 patients with a retained endotracheal tube in the PPC group (141/177, 80%) versus 110 patients (110/157, 70%) in the group without PPCs (*P* = 0.5).

Table 4 highlights the multivariate stepwise logistic regression analysis with respect to the incidence of PPCs. All parameters analyzed in the univariate analysis shown in Tables 2 and 3 were included in this multivariate approach. Only the significant risk factors are shown. Among the patient-specific parameters ASA score ≥ 3 was the only independent risk factor (OR: 1.7, 95% CI: 1.1–2.6; *P* = 0.025). There were two modifiable anesthesia-related risk factors for PPCs in this study cohort. Those were intraoperative PRBCs (OR: 1.9, 95% CI: 1.2–3.0; *P* = 0.009) and TEA (OR: 2.0, 95% CI: 1.01–3.8; *P* = 0.046).

**Table 4:** Multivariate stepwise logistic regression analysis of patient-specific and procedural risk factors with respect to PPCs

	OR (95% CI)	*P* Value
Patient-specific risk factors		
ASA ≥ 3	1.7 (1.1–2.6)	0.025
Procedural risk factors		
PRBC	1.9 (1.2–3)	0.009
TEA	2.0 (1.01–3.8)	0.046

*ASA* American Society of Anesthesiology, *PRBC* Packed red blood cell, *TEA* Thoracic epidural anesthesia

Table 5 shows the multivariate stepwise logistic regression analysis with respect to the 90-days mortality rate of patients with PPCs. All parameters analyzed in the univariate analysis shown in Tables 2 and 3 were included in this multivariate approach. Only the significant risk factors are highlighted. The results resemble those of Table 4 but with higher odds ratios for the same independent risk factors. Among the patient-specific parameters ASA score ≥ 3 was the only independent risk factor (OR: 2.6, 95% CI: 1.1–6.2; *P* = 0.036). There were two modifiable anesthesia-related risk factors for 90-days mortality of patients with PPCs in this study cohort. Those were intraoperative PRBCs (OR: 5.0, 95% CI: 2.0–12.6; *P* = 0.001) and TEA (OR: 3.9, 95% CI: 1.6–9.7; *P* = 0.003).

**Table 5:** Multivariate stepwise logistic regression analysis of patient-specific and procedural risk factors with respect to 90-days mortality of patients with PPCs

	OR (95% CI)	*P* Value
Patient-specific risk factors		
ASA ≥ 3	2.6 (1.1–6.2)	0.036
Procedural risk factors		
PRBC	5.0 (2.0–12.6)	0.001
TEA	3.9 (1.6–9.7)	0.003

*ASA* American Society of Anesthesiology, *PRBC* Packed red blood cell, *TEA* Thoracic epidural anesthesia

## Discussion

This retrospective study including 335 patients undergoing open esophagectomy for esophageal cancer is the first study examining various modifiable risk factors with regard to mortality associated with PPCs. The multivariate stepwise logistic regression analysis was used to assess the importance of procedural and potentially modifiable risk factors in the context of existing patient-specific risk factors. The main results of this study can be summarized as follows. First, the incidence of PPCs in this study cohort was 52% (175/335) associated with a 90-days mortality rate of 8% (26/335). Second, ASA score ≥ 3 was the only independent patient-specific risk factor for PPCs and the associated mortality in this study cohort. Third, this study revealed two modifiable independent risk factors for PPCs and consecutive 90-days mortality. Both anesthesia-related risk factors, intraoperative PRBCs and TEA were ranked higher than the patient-specific one according to the highlighted Odds ratios of the multivariate analysis. Although significant in the univariate approach the total amount of crystalloids was not characterized as an independent risk factor for PPCs in the multivariate analysis. Fourth, type of general anesthesia (TIVA versus gas) did not show a difference between the groups with respect to the incidence of PPCs and mortality.

The overall 30- and 90-days-mortality rate of 5 and 11% in this study cohort is in accordance with current literature [1, 24, 25]. However, this is the first study focusing on mortality related to PPCs. PPCs after esophagectomy are the most frequent postoperative complications [1, 4, 9, 26, 27]. The majority of patients who died within 90 days after surgery suffered from PPCs 70% (26/37) in our study cohort. The number of patients with PPCs 52% (175/335) in our study cohort appears higher than described in current literature (incidences of 21 to 38%) [1, 26, 27]. One reason for this discrepancy might be the fact that the definition of PPCs is heterogenous. The following PPCs were included in this study: pneumonia, pleural effusion, re-intubation, tracheostomy, chylothorax and pleural empyema. The incidence of pleural effusion in our study cohort is quite high with an incidence of 31%. Pleural effusion is rarely described as a PPC after esophagectomy. However, due to its consecutive respiratory impairment and possible side effects of prolonged thoracic drainage, pleural effusion was added as a PPC.

Our results with respect to patient-specific risk factors for postoperative complications are partially in accordance with current literature. The only independent patient-specific risk factor in this study cohort was ASA score ≥ 3 which was also described by several authors [8, 16, 26]. Further patient-specific risk factors like age, smoking, gender, neoadjuvant radio−/chemotherapy or preoperative anemia were not confirmed in this retrospective study [1, 5, 7, 9, 16, 19, 25].

Among the procedural parameters the surgery-related risk factors did not show any significant results, although blood loss and transthoracic approach were described as independent risk factors for adverse outcome with respect to postoperative mortality and PPCs after esophagectomy [1, 5]. A reason for this discrepancy with respect to the surgical approach might be the low number of bench mark patients who underwent transhiatal esophagectomy 8% (26/335) in this study cohort.

Among the procedural parameters the anesthesia-related risk factors are divided into three subgroups for the discussion section. These are intraoperative fluid therapy including quality and quantity of fluids, the type of general anesthesia (TIVA versus gas) and the use of perioperative TEA.

Several studies emphasized the importance of intraoperative restrictive fluid therapy during esophagectomy to reduce the incidence of PPCs [10, 11]. Although, patients of the PPC group received significantly more intraoperative crystalloid fluids, this difference was no longer obvious in the multivariate logistic regression analysis. The preoperative hemoglobin level and the intraoperative blood loss did not show a difference between patients with and without PPCs, nevertheless patients of the PPC group received PRBCs more often. This result was verified in the multivariate stepwise logistic regression analysis where the intraoperative transfusion of PRBCs was an independent risk factor for PPCs and its associated 90-days mortality. This is in accordance with current literature where liberal transfusion of PRBCs during esophagectomy was described as a risk factor for postoperative morbidity with respect to general postoperative complications, PPCs, increased 30-days mortality and worse long-term outcome [13, 28–31]. To our best knowledge there is no other study that focused on the 90-days mortality related to PPCs in esophageal cancer patients after esophagectomy. In our study the intraoperative use of colloids or fresh frozen plasma was not associated with an increased risk for PPCs or postoperative mortality. Subramanian and colleagues described the intraoperative use of FFP as an independent risk factor for major postoperative infectious complications including pneumonia after esophagectomy [32]. Data on the intraoperative use of colloids during esophagectomy and its effects on postoperative morbidity and mortality are scarce. Ahn and colleagues examined the effect of hydroxyethyl starch (HES) on postoperative renal function in thoracic surgery [33]. They concluded that HES should be administered with caution especially in high-risk patients undergoing thoracic surgery. Although this study focused on the incidence of acute kidney injury (AKI), they also mentioned an increased rate of PPCs in the group of patients with AKI who received more HES than the group without AKI. Further studies are needed to draw reliable conclusions on this topic.

Due to the fact that the type of anesthesia maintenance (intravenous versus gas) during esophagectomy influences the inflammatory cytokine production in the airway epithelium and impairs the pulmonary circulation, Zhang and colleagues hypothesized that the incidence of postoperative pneumonia is also effected [34–36]. However, there was no difference with respect to the incidence of postoperative pneumonia [36]. This is in accordance with our results. The type of general anesthesia did not show a difference between patients with and without PPCs and was not characterized as an independent risk factor for PPCs or 90-days mortality of patients with PPCs in our study cohort. Admittedly, the comparison of our results with current literature on this topic appears difficult because instead of sevoflurane which was mostly used in past studies, the majority of our patients received isoflurane or desflurane.

Data on the use of TEA to reduce the incidence of PPCs after esophagectomy are inconsistent [2, 14, 16–18, 20]. The current meta-analysis by Visser and colleagues shows no significant benefit for TEA compared to systemic analgesia with respect to PPCs or postoperative pain scores at 24 and 48 h after esophagectomy. In contrast to these results several authors published a significant reduction of the incidence of postoperative pneumonia by using TEA in patients undergoing esophagectomy [14, 15, 20]. Zingg and colleagues were not able to confirm these results with respect to postoperative pneumonia, but showed a significant decrease of postoperative respiratory failure and ARDS [16]. Esophageal cancer patients with TEA after esophagectomy showed decreased opioid consumption and reduced duration of ICU stay [19]. To our best knowledge there are no results on the positive effect of TEA with respect to the 90-days mortality related to PPCs after esophagectomy. The univariate analysis of the subgroup of patients with an ASA score ≥ 3 shows that especially high risk patients (ASA score ≥ 3) undergoing thoracotomic esophagectomy benefit from epidural analgesia.

For each patient only the daily maximum pain score at rest was used for the final analysis. For postoperative pain assessment the pain level at coughing and moving appears more valid to assess a sufficient pain therapy. Due to the retrospective character of this study, data on the pain score during respiratory therapy was not available as these data were not recorded in clinical routine. It was also not documented whether patients were able to participate sufficiently in physiotherapy with respect to their state of consciousness under opioid therapy. Therefore the comparison of pain scores at rest might appear misleading.

Our results show that the absence of TEA for patients undergoing open esophagectomy is a major risk factor for PPCs and the 90-days mortality related to PPCs.

## Conclusion

In esophageal cancer patients undergoing thoracotomic esophagectomy epidural analgesia and the avoidance of intraoperative blood transfusion are significantly associated with a reduced 90-days mortality related to PPCs.

## Data Availability

The datasets generated and analyzed during the current study are available from the corresponding author on reasonable request.

## Abbreviations

AKI: Acute kidney injury
ARDS: Acute respiratory distress syndrome
ASA: American society of anesthesiology
BMI: Body-mass-index
DLCO: Diffusion capacity of the lung for carbon monoxide
FEV_1_: Forced expiratory volume in 1 second
FFP: Fresh frozen plasma
HES: Hydroxyethyl starch
ICU: Intensive care unit
PPC: Postoperative pulmonary complications
PRBC: Packed red blood cells
SLV: Single-lung ventilation
TEA: Thoracic epidural anesthesia
TIVA: Total intravenous anesthesia
UICC: Union Internationale Contre Cancer
